# Increased reflux secondary bile acids are associated with changes to the microbiome and transcriptome in Barrett’s esophagus

**DOI:** 10.1080/19490976.2025.2545420

**Published:** 2025-08-22

**Authors:** Ceylan Tanes, Yun Li, Gary W. Falk, Gregory G. Ginsberg, Kenneth K. Wang, Prasad G. Iyer, Charles J. Lightdale, Armando Del Portillo, Stephen M. Lagana, Timothy C. Wang, Anil K. Rustgi, Michael Quante, Zhezhen Jin, Gary D. Wu, Elliot S. Friedman, Kyle Bittinger, Hongzhe Li, Julian A. Abrams

**Affiliations:** a Division of Gastroenterology, Hepatology and Nutrition, Children’s Hospital of Philadelphia, Philadelphia, PA, USA; b Department of Biostatistics, Epidemiology and Informatics, University of Pennsylvania Perelman School of Medicine, Philadelphia, PA, USA; c Division of Gastroenterology and Hepatology, University of Pennsylvania Perelman School of Medicine, Philadelphia, PA, USA; d Division of Gastroenterology and Hepatology, Mayo Clinic, Rochester, MN, USA; e Division of Gastroenterology and Hepatology, Mayo Clinic, Scottsdale, AZ, USA; f Division of Digestive & Liver Diseases, Columbia University Irving Medical Center, New York, NY, USA; g Herbert Irving Comprehensive Cancer Center, Columbia University, New York, NY, USA; h Department of Pathology and Cell Biology, Columbia University Irving Medical Center, New York, NY, USA; i Digestive and Liver Disease Research Center, Columbia University Irving Medical Center, New York, NY, USA; j Department of Medicine II, University of Freiburg, Freiburg, Germany; k Department of Biostatistics, Mailman School of Public Health, Columbia University, New York, NY, USA

**Keywords:** Barrett’s esophagus, esophageal adenocarcinoma, bile acids, microbiome

## Abstract

Bile acids are a major component of gastro-esophageal refluxate, thought to contribute to the development of Barrett’s esophagus (BE) and esophageal adenocarcinoma (EAC). As the microbiome shifts with EAC progression and bile acids influence bacterial composition, we examined these connections in a multi-center, cross-sectional study. We analyzed biospecimens from patients undergoing endoscopy using LC-MS to quantify bile acids in gastric aspirates, 16S rRNA sequencing for tissue microbiome profiling, and RNA sequencing on BE or cardia tissue. Among 153 patients (52 controls, 101 BE: 50 no dysplasia, 10 indefinite, 17 low-grade dysplasia, 17 high-grade dysplasia, and 7 EAC), we observed increased *Streptococcus* in BE tissue; dysplasia and EAC were associated with more *Lactobacillus* and decreased *Actinomyces* and other genera. Refluxate bile acids were mainly conjugated, indicating minimal bacterial metabolism, while BE patients had elevated secondary bile acid levels. *Streptococcus* correlated with upregulation of *IL6*, *FGF2*, and *HGF*, and decreased *Actinomyces* showed the most associations with gene expression, including the oxidative phosphorylation pathway. We identified two distinct BE gene expression clusters independent of histology, bile acid, or microbiome composition. These findings suggest bile acids shape the BE microbiome and associate with gene expression changes potentially relevant to EAC development.

## Introduction

Esophageal adenocarcinoma (EAC) is associated with a poor prognosis and continues to represent a major public health burden in Western countries.^1^ Barrett’s esophagus (BE) is thought to be the precursor lesion to EAC, and gastro-esophageal reflux disease (GERD) is the strongest modifiable risk factor for both BE and EAC. The distal esophageal lumen is exposed to a variety of factors including refluxate from the stomach as well as bacteria on the tissue surface, yet relatively little is known regarding the details of these exposures and how they may contribute to the development of EAC.

Bile acids comprise a major component of refluxate from the stomach, and there is an extensive body of experimental data to suggest that bile acids can have procarcinogenic effects in the esophagus. However, the biological effects of distinct bile acids vary; for example, the secondary bile acid deoxycholic acid promotes colonic neoplasia, whereas ursodeoxycholic acid has anti-inflammatory properties.^2,3^ In the L2-IL1B mouse model of BE/EAC, administration of deoxycholic acid accelerates the development of neoplasia.^4^ In humans, however, whether distinct bile acids (primary vs. secondary, conjugated vs. unconjugated) have differing effects on the development of EAC are unknown.

In the small intestine and colon, bacteria and bile acids are closely linked. Bacteria are responsible for much of bile acid metabolism, including bile acid deconjugation via bile salt hydrolase as well as the conversion of primary to secondary bile acids, and bile acids can shape bacterial composition via bactericidal and other effects.^5^ In the L2-IL1B model, mice raised in a germ-free setting have reduced development of BE and dysplasia,^6^ suggesting that bacteria are important co-factors in the development of EAC. While microbiome alterations have been described in BE and EAC,^7–9^ any biological relationships between bacteria and esophageal neoplasia is unknown. Further, inter-relationships between esophageal bacterial composition and bile acids have not been elucidated.

To gain additional insights into these critical gaps in knowledge, we performed a comprehensive assessment of the microbiome, reflux bile acids, and esophageal tissue gene expression in patients with and without BE and associated dysplasia or EAC.

## Methods

### Study design

Patients with and without BE who underwent upper endoscopy for clinical indications at Columbia University Irving Medical Center, Mayo Clinic-Rochester, and University of Pennsylvania were prospectively enrolled from February 2018 through September 2021. Patients were ≥18 y old, and for BE patients, had histologically confirmed BE ≥2 cm in length and took proton pump inhibitors (PPIs) at least daily for 3 months prior to enrollment. Patients without BE were enrolled stratified 1:1 based on current PPI use. Additional eligibility criteria as well as data recorded are provided in *Supplementary Methods*.

### Biospecimen analyses

Bile acids were profiled from gastric aspirate samples by liquid chromatography-mass spectrometry. Microbiome profiling was performed of saliva, oral rinse, esophageal squamous brushings, and BE (or cardia from controls) brushings. This was done by 16S rRNA gene sequencing of the V1-V2 hypervariable regions. DNA was extracted from samples and libraries annealing to the V1-V2 region of the 16S rRNA gene were generated to be sequenced on Illumina MiSeq. Sequence data were processed using QIIME2 version 2019.7.^10^ Tissue gene expression analyses of BE and cardia were performed by bulk RNA sequencing (RNA-Seq) using Illumina NovaSeq 6000 using paired-end 100bp chemistry. Reads were mapped to the human transcriptome (GRCh38) using the pseudoalignment software kallisto (0.44.0).^11^ Analyses were restricted to those with RNA Integrity Number (RIN) values greater than 5. See *Supplementary Methods* for additional details related to biospecimen processing and analyses.

### Statistical analyses

#### Microbiome

Data files from QIIME were analyzed in the R environment for statistical computing. Global differences in community composition were visualized using Principal Coordinates Analysis. Community-level differences between sample groups were assessed using the PERMANOVA test.^12^ The relative abundances of the ASVs that were assigned to the same taxon were summed. Effect of Barrett’s status and PPI use on log transformed taxon abundances was assessed using linear models. While recent antibiotics use was an exclusion criterion for the study, 13 patients had received antibiotics within 3 months of sample collection. We addressed this by including antibiotics exposure as a covariate. Unless PPI use was tested for directly, it was also added as a covariate. To further assess any effects of antibiotics exposure or statin use, we performed sensitivity analyses removing the subjects who had received antibiotics within the prior 3 months or adding statin use as a covariate and repeating the differential abundance tests. Only the taxa with at least 0.5% mean relative abundance in at least one sample type were tested. When multiple tests were conducted, we adjusted for false discovery rate (FDR) using Benjamini-Hochberg method.

#### Power analysis

With our study sample size of 52 controls and 101 subjects with BE, we had 80% power to detect a 0.48 SD difference in Shannon index between study groups. Furthermore, considering the 24 most abundant taxa, our sample size had 80% power to detect a 0.64 SD difference in relative abundance with Bonferroni-adjusted *p* < 0.1 between groups.

#### Bile acids

Levels of individual bile acids as well as total levels of primary or secondary conjugated bile acids were tested between study groups or across dysplasia levels using linear models with PPI use as a covariate. Spearman correlation was used to associate bile acid levels and relative abundance of total Gram positive and negative bacteria.

#### Gene expression

The statistical algorithm based on the negative binomial generalized linear model, DESeq2 (implemented in R programming language),^13^ was used for the two groups comparison and the regression analysis. The model was adjusted for underlying histology as well as risk factors for EAC, including age, BMI, sex, history of GERD, smoking history (ever/never), and family history of BE/EAC. K-means unsupervised clustering was performed based on the variance stabilizing the transformed values. The pathway enrichment analysis was performed using Fisher’s exact test. The background genes were selected based on the mean of normalized counts, the R function genefinder (from package genefilter) was used and for each of the significant genes (FDR < 0.1), 10 background genes with similar pattern of expression were selected. The reference database including 186 Kyoto Encyclopedia of Genes and Genomes (KEGG) canonical pathways was used to identify significantly altered pathways (FDR < 0.1).^14^ Regression analysis of gene expression with bile acids and bacterial relative abundance levels was conducted using DESeq2.

## Results

### Overview of the study cohort

A total of 166 patients were enrolled in the study, 153 were eligible for inclusion in the study analyses (Supp Figures S1 and S2); there were 52 non-BE controls and 101 patients with BE (50 ND, 10 IND, 17 LGD, 17 HGD, 7 EAC). All EAC patients had intramucosal adenocarcinoma. The mean age was 60.3 (SD 14.0), 65% were male, 97% were white, and 98% were non-Hispanic. Compared to controls, those with BE were older, and a higher proportion were male, on statins, ever-smokers, and had a family history of BE or EAC (Table 1). The most common indications for upper endoscopy among the control patients were abdominal pain/dyspepsia (*n* = 22), and GERD (*n* = 18), followed by celiac disease, anemia, and diarrhea (Supp Table 1).

**Table 1.** Characteristics of study participants.

	Non-BE (n = 52)	BE (n = 101)	P-value*
***Demographics***			
Age, years: mean (SD)	50.5 (±15.5)	65.4 (±10.1)	< 0.001
BMI: mean (SD)	29.7 (±6.9)	30.2 (±5.4)	0.3
Waist to hip ratio (SD)	0.97 (±0.097)	1.00 (±0.11)	0.2
Male sex: n (%)	22 (42%)	78 (77%)	< 0.001
Non-Hispanic ethnicity: n (%)	52 (100%)	98 (97%)	0.6
White race: n (%)	50 (96%)	99 (98%)	0.6
PPI use: n (%)	27 (52%)	101 (100%)	< 0.001
Aspirin use: n (%)	16 (31%)	38 (38%)	0.5
Statin use n (%)	12 (23%)	53 (52%)	< 0.001
Reflux n (%)	34 (65%)	94 (93%)	< 0.001
Ever smoker: n (%)	19 (37%)	60 (59%)	0.01
Family history of BE or esophageal cancer: n (%)	4 (8%)	25 (25%)	0.02
***Endoscopic characteristics***			
Hiatal hernia size: median (IQR)	0 (IQR 0–0)	3 (IQR 2–4)	< 0.001
BE length (C): median (IQR)	N/A	1 (IQR 0–4)	
BE length (M): median (IQR)	N/A	4 (IQR 2–7)	

*Wilcoxon or Fisher exact p-values reported for difference between controls and BE.

### The esophageal microbiome is altered in Barrett’s esophagus

**Figure 1.**
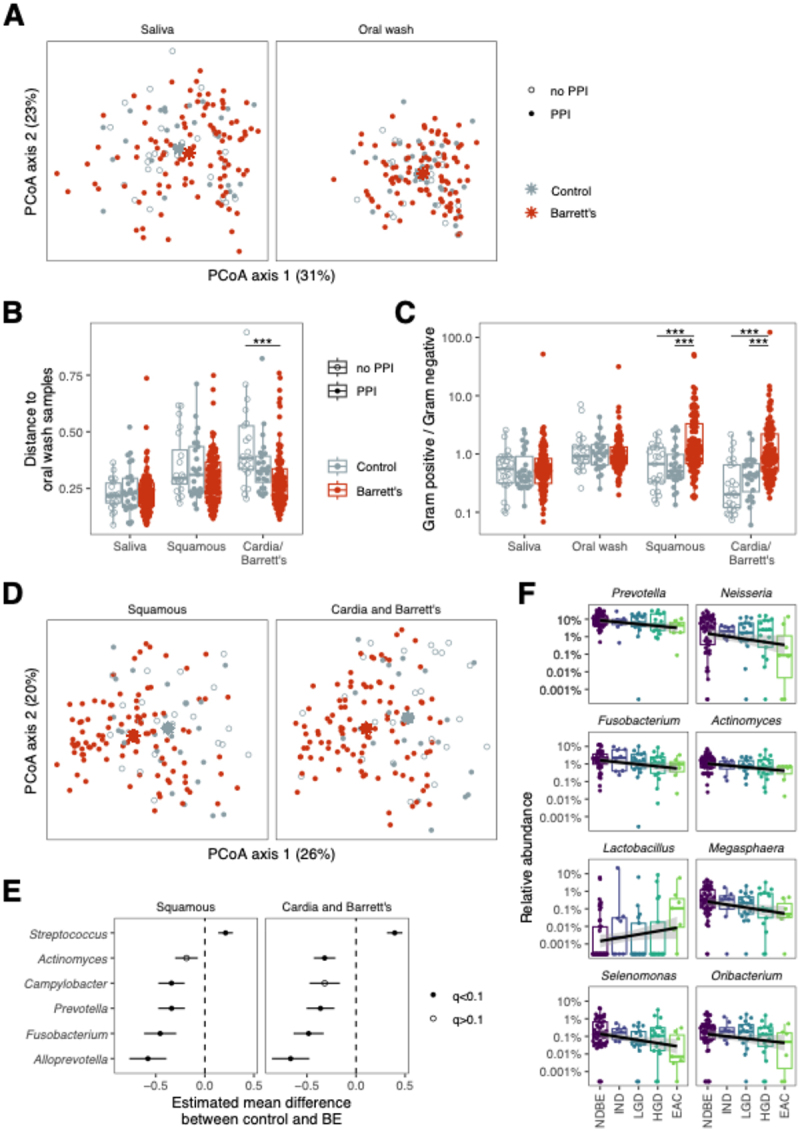
Patients with Barrett’s esophagus have a distinct tissue-associated microbiome. A) Principal coordinates analysis (PCoA) of weighted UniFrac distances show no evidence of significant clustering comparing BE with non-BE in saliva (left) and oral swash (right); B) the BE tissue microbiome was more closely related to oral wash within individuals as compared to controls off of PPIs (*p* < 0.001) and non-significantly compared to controls on PPIs (*p* = 0.07). Shown are within-individual weighted UniFrac distances compared to oral wash; C) the gram positive:negative ratio was higher in BE tissue compared to cardia in controls (vs. PPI users, *p* < 0.001; vs. PPI non-users, *p* < 0.001); D) PCoA of weighted UniFrac distances for squamous and BE/cardia tissue demonstrating significant differences in beta diversity comparing BE with controls (controlled for PPI use and antibiotics; squamous, PERMANOVA *p* = 0.003; BE/cardia, PERMANOVA *p* = 0.001); E) BE patients had significant differences in the relative abundance bacterial genera compared to controls in both squamous and BE tissue; F) bacterial genera with significantly increased and decreased relative abundance associated with stages of progression from BE to EAC. *p < 0.05, **p < 0.01, ***p < 0.001.

We performed 16S rRNA gene sequencing from the cohort of 152 patients on the following samples: oral wash, saliva, esophageal squamous brushings, BE (or cardia in controls) brushings. Comparing BE and control patients, there were no differences in the alpha diversity of the oral microbiome. PERMANOVA test on weighted UniFrac distances showed no separation of centroids between patients with BE and controls regardless of PPI use (Figure 1(A)). We then assessed intra-individual relationships among the various sampling sites. The UniFrac distance between oral wash and BE/cardia was greater in non-PPI controls compared to BE patients, and there remained an attenuated difference comparing PPI controls to BE patients (Figure 1(B)). The overall Gram positive:Gram negative ratio progressively decreased with more distal sampling in non-PPI controls; this decrease was attenuated in PPI controls and increased slightly in BE patients (Figure 1(C)). In sum, these data suggest that oral and esophageal bacterial composition are more closely related in BE patients compared to those without BE, and that this is explained partly, but not fully, by PPI use.

We then analyzed the esophageal squamous and BE/cardia microbiome in BE and control patients. We first compared the bacterial community structures between BE patients and controls using Principal Coordinate Analysis on weighted UniFrac distances (Figure 1D). Even after correcting for antibiotic and PPI use, the microbiome was distinct in BE patients compared to controls in both squamous (*p* = 0.003) and BE/cardia brushings (*p* = 0.001). After stratifying controls by PPI use, these differences between BE and control patients on PPIs were attenuated but persisted, indicating that PPI use alone did not explain the observed changes in the lower esophageal microbiome of BE patients.

We next investigated if there were specific bacteria that accounted for the community level differences between BE and control patients (Figure 1(E)). *Streptococcus* was the only genus with higher levels in BE patients in both squamous and Barrett’s brushings. Conversely, the levels of *Prevotella*, *Alloprevotella* and *Fusobacterium* were lower in BE patients in both squamous and Barrett’s brushings, and *Actinomyces* was lower only in Barrett’s brushings. In sensitivity analyses, we excluded the 13 patients who had received antibiotics within the prior 3 months and found no qualitative differences; differences in *Streptococcus*, *Fusobacterium* and *Alloprovotella* were still statistically significant in both squamous and cadia/Barrett’s samples, and *Actinomyces*, *Campylobacter*, and *Prevotella* maintained that same degree of association but were no longer significant. When added stain use as a covariate, all the genera remained statistically significantly different with the exception of *Actinomyces* (q = 0.104). We then looked for bacteria in Barrett’s brushings that correlated with stages of progression from BE to EAC. We found an increase in *Lactobacillus* with progressive stages of neoplasia, as has been reported previously.^15^ We also observed decreasing linear trends for seven genera with progressive stages of neoplasia: *Actinomyces*, *Prevotella*, *Neisseria, Fusobacterium, Megasphaera, Selenomonas*, and *Oribacterium* (Figure 1(F)). After excluding the subjects who had received antibiotics within the prior 3 months, all genera but *Lactobacillus* and *Megasphaera* remained significantly correlated with progressive stages of neoplasia, but the direction of correlation for the two genera remained unchanged. When we added statin use as a covariate, all but *Actinomyces* levels were still correlated with progressive stages of neoplasia, although the direction of correlation was also consistent for *Actinomyces* levels compared with the initial analysis.

### Refluxate in Barrett’s esophagus patients has increased conjugated secondary bile acids

**Figure 2.**
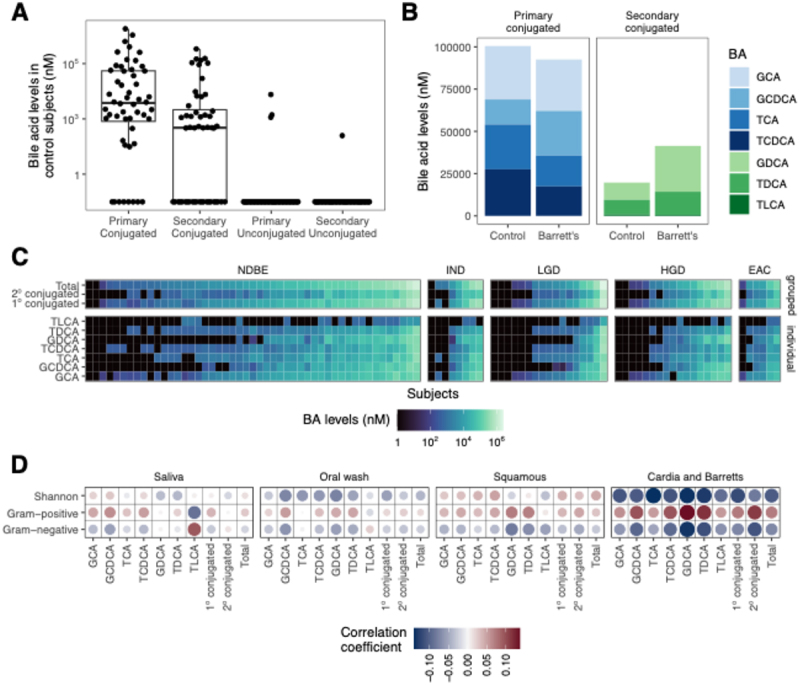
Secondary bile acid levels are increased in refluxate in patients with Barrett’s esophagus. A) virtually all of the measure bile acids were primary and secondary conjugated bile acids; B) patients with BE had significantly greater secondary bile acids compared to controls; C) there were no significant differences in bile acid composition across stages of BE to EAC; D) in BE/cardia tissue, there were non-significant trends towards positive correlations between bile acid levels and gram-positive bacteria and inverse correlations with gram-negative bacteria. The bile acid:bacteria correlations diminished with more proximal sampling sites.

We characterized the gastric aspirate bile acid levels in our study cohort (*n* = 149) as a surrogate for refluxate bile acid composition. The overwhelming majority of bile acids were conjugated; each of the deconjugated bile acids was detected in <5% of patients (Figure 2(A)). The near absence of deconjugated bile acids suggests that in the stomach there is little bacterial metabolism via bile salt hydrolase, the first step in bile acid modification after secretion into the intestinal tract. Within the conjugated bile acids, the levels of the secondary conjugated bile acids (*p* = 0.03) were higher in BE patients compared to controls, TDCA being the main contributor of the difference (*p* = 0.02, Figure 2(B)). There were no demographic or clinical factors associated with bile acid composition. Among controls, there were no differences in bile acid levels comparing those who were and were not taking PPIs. Interestingly, among the BE patients we found no association between bile acid levels and stages of dysplasia (Figure 2(C)).

We then assessed within-patient correlations between bile acid levels with aggregate relative abundance of Gram-positive and -negative bacteria as well as the Shannon diversity metric. There was a trend toward positive correlations between bile acid levels and Gram-positive bacteria and negative correlations with Gram-negative bacteria and diversity in BE/cardia brushings, although all the FDR values were above the 0.1 threshold (Figure 2(D)). Interestingly, controls on and off PPIs had opposite directions of correlations between bile acid levels and Gram-positive and -negative bacteria in the cardia, although none of these correlations were statistically significant (Supp Figure S3A). We further assessed relationships between bile acid levels and individual bacterial genera and found positive correlations between *Streptococcus* in BE/cardia brushings and GDCA and negative correlations between *Campylobacter* and numerous bile acids (Supp Figure S3B). In general, the correlation coefficients were greatest in BE/cardia, with diminishing strength of association with more proximal sampling. In sum, these data suggest that patients with BE have a distinct refluxate bile acid profile and that these bile acids help shape the distal esophageal microbiome, with likely only a minor effect of bacteria on bile acid composition.

### Gene expression clusters in Barrett’s esophagus

**Figure 3.**
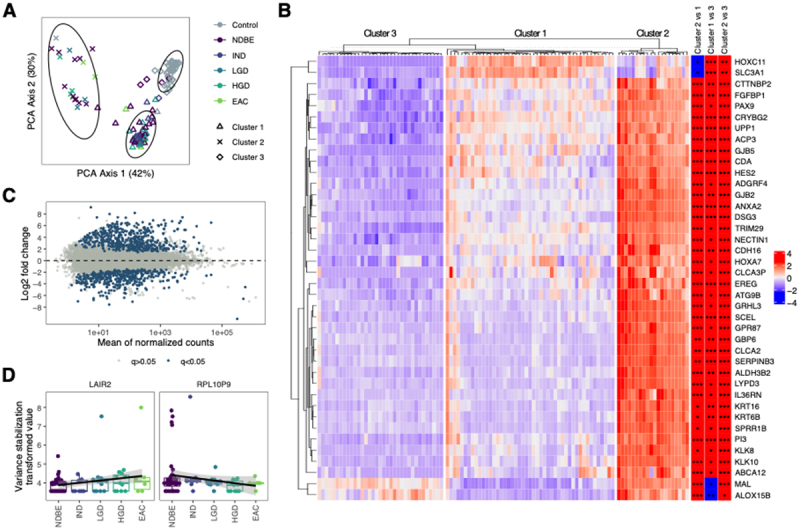
Gene expression changes in patients with BE. A) unsupervised clustering analyses demonstrated three distinct clusters, one comprised of controls and two BE clusters independent of associated histology; (B) heatmap of 40 genes that were significantly differentially expressed in all pairwise comparisons between clusters. C) Plot demonstrating large number of differentially expressed genes in BE, including numerous markers of intestinalization; D) among patients with BE, *LAIR2* was significantly increased and *RPL10P9* significantly decreased across stages from BE to EAC (analyses adjusted for clinical risk factors for EAC). NDBE: non-dysplastic Barrett’s esophagus; IND: indefinite for dysplasia; LGD: low grade dysplasia; HGD: high grade dysplasia; EAC: esophageal adenocarcinoma. * *p*<0.05, ** *p*<0.01, ***p<0.001.

We performed bulk RNA-Seq analyses of BE tissue and cardia from controls from a subset of 109 patients (control = 37, BE = 72 with RIN > 5). We initially performed k-means clustering analyses and observed three distinct clusters, two separate BE clusters (Clusters 1 and 2) and one comprised almost exclusively of control patients (Cluster 3, Figure 3(A)). Interestingly, there was no difference in the distribution of BE-associated histology in Clusters 1 and 2 (Supp Table S2). BE patients in Cluster 1 had longer BE segments compared to Cluster 2; otherwise, there were no differences in clinical characteristics between the two clusters.

Comparing the two BE clusters, Cluster 2 had increased expression of >900 genes including numerous Wnt- (*PORCN*, *WNT3A*, *WNT4*, *WNT5A*, *WNT7A*, *WNT7B*, *WNT10A*, *WNT10B*, *FZD10*) and Notch-related genes (*NOTCH3, JAG2*, *ADAM11*, *ADAM23*, *HES2*). We then further analyzed differentially expressed genes between the two clusters, restricted to the 40 genes that were also differentially expressed compared to cardia from controls (Figure 3(B)). In Cluster 1 we found increased expression of *HOXC11* and *SLC3A1* and downregulation of *MAL* and *ALOX15B*. Of the remaining 36 genes, Cluster 2 had increased expression, including upregulation of cell cycle pathways and of genes *ANXA1, KLK8, KLK10*, and *HES2*. A previous publication by Guo et al. analyzed gene expression in EAC and also noted two distinct clusters.^16^ Interestingly, we re-analyzed our data from BE patients restricted to the list of differentially expressed genes from those EAC clusters and closely reproduced the findings from the study by Guo et al. (Supp Figure S4)

We then compared differential gene expression of BE patients and controls, adjusting for key clinical covariates (age, sex, BMI, GERD, smoking history, and family history of BE/EAC). As expected, gene markers of an intestinal phenotype (*MUC2*, *TFF3*, *MUC17*, *CDX2*, *FABP1*, *FABP2*) were upregulated in BE patients (Figure 3(C)). KEGG pathway analyses identified enrichment in drug metabolism, arginine and proline metabolism, and PPAR signaling in BE.

We also investigated genes with altered expression in BE patients and associated with stages of neoplasia from no dysplasia to EAC. After adjusting for clinical covariates, we identified only two genes with altered expression with progressive stages of neoplasia: increased *LAIR2* and decreased *RPL10P9*. (Figure 3(D)) *LAIR2* encodes leukocyte-associated immunoglobulin-like receptor 2, which plays a role in anti-tumor immunity and may represent a Treg and exhausted T cell marker in certain solid tumors.^17,18^ *RPL10P9* encodes ribosomal protein L10 pseudogene 9, and there is only limited data regarding its function.

We then assessed whether different genes may be associated with progressive stages of neoplasia in the two BE clusters, again adjusting for clinical covariates (Supp Table S3). In Cluster 1, we found no genes associated with progressive stages of neoplasia with an FDR < 0.05; however, we observed decreased expression of *DSC3* and *GPR87* with an FDR < 0.10. *DSC3*, which encodes desmocollin-3, is a p53 target gene and has decreased expression in the setting of mutant p53.^19,20^ *GPR87* encodes G protein coupled receptor 87, which is essential for p53-dependent cell survival.^21^ In Cluster 2 we found 15 genes with altered expression (FDR < 0.05) including increased expression of the oncogene *FOSB*, the T cell activator *TAGAP*, and the lymphocyte homing receptor *ITGA4*.

When we compared gene expression of Clusters 1 and 2 in BE patients, we found no differentially abundant bacterial taxa and no differences in bile acid levels. In sum, these findings suggest that these two clusters are not only independent of underlying histology but also of certain major environmental exposures, possibly indicating that there are two distinct and intrinsic pathways to progress from BE to EAC, with differences related to Wnt and Notch pathway activity as well as inflammation and immune cell signaling.

### Relationships between bile acids, the microbiome, and the transcriptome in BE

**Figure 4.**
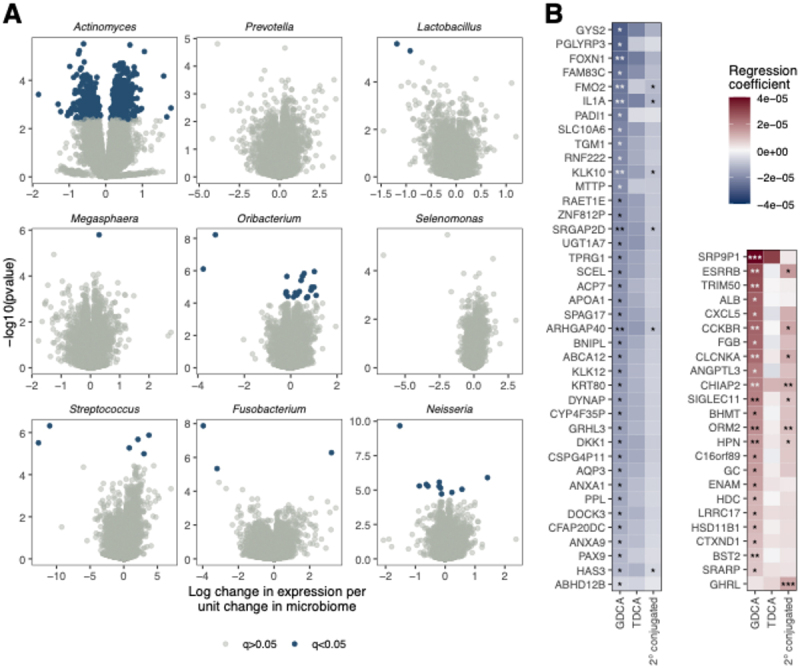
Relationships between the microbiome and bile acids with tissue gene expression. A) Volcano plots demonstrating significant associations between relative abundance of bacterial genera associated with BE progression to EAC and tissue gene expression; B) up- and down-regulated genes associated with the secondary bile acids GDCA and TDCA and with total conjugated secondary bile acids.

We then investigated potential relationships between the lower esophageal microbiome and tissue gene expression. We first analyzed the nine bacterial genera with higher relative abundance in BE compared to controls and/or with statistically significant correlation with progressive stages of esophageal neoplasia (using DESeq2), again adjusting for key clinical covariates and associated histology (Figure 4(A)). *Streptococcus*, which was increased in BE, positively correlated with expression of *IL6* (FDR < 0.1) as well as growth factors *FGF2* and *HGF* (FDR < 0.1). *Lactobacillus*, increased with progressive stages of neoplasia, was inversely associated with *TRIM50*, an E3 ubiquitin ligase that inhibits the Myc signaling pathway and is downregulated in gastric cancer.^22^ *Actinomyces* relative abundance was associated with the greatest number of changes in the tissue transcriptome, with altered expression of >500 genes. In pathway analyses, the decreased relative abundance of *Actinomyces* observed with progressive stages of neoplasia correlated with upregulation of oxidative phosphorylation pathway (FDR < 0.001). Decreased *Oribacterium* was associated with upregulation of DNA replication and cell cycle pathways (Fisher’s exact test, FDR < 0.1). Decreases in *Neisseria* relative abundance associated with increased expression of *TNFSF11* (a.k.a. *RANKL*), a NFκB ligand that is increased in BE-associated HGD and EAC.^23^ Taken together, these findings suggest that lower esophageal microbiome alterations in BE-associated dysplasia and EAC associate with inflammatory and other pro-neoplastic changes to gene expression.

We then assessed associations between refluxate bile acid levels and gene expression for those bile acids increased in BE (total conjugated secondary bile acids, GDCA, TDCA). (Figure 4(B)) We observed the greatest number of associations with GDCA, with notable positive correlations with *CXCL5*, the gastrin receptor *CCKBR*, and histidine decarboxylase (*HDC*). Interestingly, increased GDCA was associated with decreased expression of genes typically expressed in esophageal squamous tissue such as *KRT80*, *IL1A* and *GRHL3* and downregulation of the keratinocyte differentiation pathway (Fisher’s exact test for GDCA using GO biological process). To evaluate the potential effects of PPI use on associations between bile acids and transcriptome, we repeated the correlation analysis including the control subjects with PPI use and study group as covariates. Out of the 77 initial correlations that we reported, 76 maintained the same direction of correlation and 23 remained statistically significant. Furthermore, in addition to the correlation between *CXCL5* and GDCA, we identified correlations with GCDCA and total conjugated secondary bile acids. Additionally, there were new significant correlations identified including markers of inflammation (e.g. *ECRG4*) and genes associated with a variety of epithelial cancers (*PEBP4*, *CUX2*, *ESRRG*, *FGG*).

## Discussion

In this cross-sectional study of Barrett’s esophagus and EAC, we performed comprehensive relational analyses of refluxate bile acids, the upper aero-digestive microbiome, and BE tissue gene expression to gain insights as to how these factors may be important to the development of EAC. Compared to non-BE, BE patients had marked differences in all three components. However, smaller differences were noted in dysplasia or EAC compared to no dysplasia.

There were significant differences in quantitative refluxate bile acid composition in BE patients, not only in terms of total bile acids but also notably in conjugated forms of DCA. Bile acids have pro-inflammatory and carcinogenic properties in BE, inducing inflammation and causing oxidative stress and DNA damage.^24–27^ DCA causes inflammation and apoptotic resistance^28^ and induces Notch signaling and accelerates neoplasia in the L2-IL1B mouse model of BE and EAC.^4^ Further, our group recently demonstrated that secondary bile acids promote esophageal neoplasia via FXR antagonism.^29^ Interestingly, in pathway analyses increased levels of conjugated forms of DCA associated with decreased keratinocyte differentiation, with downregulation of numerous genes typically expressed in esophageal squamous tissue. This raises the possibility that bile acid exposure in the esophagus shifts the balance to favor a columnar rather than a squamous phenotype in the setting of reflux-induced injury.

Refluxate bile acids were present almost exclusively in the conjugated form. Bile acids are secreted in the conjugated form and then deconjugated by intestinal bacteria expressing bile salt hydrolase,^30^ and thus the near absence of unconjugated bile acids suggests that minimal bacterial metabolism of bile acids occurs in the stomach. We did find diminishing correlations between refluxate bile acid levels and bacterial composition with more proximal sampling sites, providing at least indirect evidence that reflux bile acid exposure may help shape the lower esophageal microbiome. Further, the fact that controls on and off PPIs had opposite correlations between bile acid levels and microbiome features suggests that pH may be an important mediator of the impact of bile acids on bacterial composition.

BE patients had several alterations in the lower esophageal microbiome, including increased *Streptococcus* and decreased *Actinomyces, Prevotella*, *Alloprevotella* and *Fusobacterium*. PPI use attenuated but did not eliminate these associations, again suggesting that refluxate pH is an important modifier but not the sole determinant of esophageal microbiome composition. The findings could have several but not mutually exclusive explanations. BE patients had increased refluxate bile acids, which likely contributed to lower esophageal microbiome differences in BE and control patients. However, even compared to control patients on PPIs, the intra-individual relationship between the oral and esophageal microbiome in BE patients was stronger than in control patients (even on PPIs), suggesting that the BE-associated tissue microbiome may be more resistant to local perturbations by bile acids. Barrett’s epithelium and microenvironment may promote a distinct microbiome; however, similar alterations were seen in squamous brushings, suggesting that the mere presence of BE less likely determines lower esophageal microbiome composition, and perhaps there are unaccounted patient-level factors contributing to the observed differences.

There were several bacterial taxa with altered relative abundance in the lower esophagus associated with stages of progression to EAC. *Actinomyces* had decreasing relative abundance and associated with upregulation of oxidative phosphorylation pathway gene expression. *Actinomyces* may play an important role in both dietary and salivary nitrate metabolism via conversion to nitrous oxide instead of pro-inflammatory nitrites.^31^ Decreased relative abundance of *Oribacterium* was associated with significant upregulation of DNA replication and cell cycle pathways. *Lactobacillus* was the only genus significantly increased across neoplastic stages, and high abundance of *Lactobacillus* has previously been described in EAC tumors.^15^ *Streptococcus*, the most abundant genus in the upper aero-digestive tract, was increased in BE and was associated with increased expression of growth factors *FGF2* and *HGF* as well as *IL6*, a pro-inflammatory cytokine that likely plays a key role in promoting esophageal neoplasia.^4^ These findings are all consistent with a potential mechanistic role for bacteria in the development of EAC.

We found two distinct gene expression clusters in BE patients independent of associated histology, suggesting two distinct pathways for progression to EAC. Cluster 2 was characterized by upregulation of numerous Notch and Wnt related genes; these pathways are key to maintaining intestinal homeostasis, and our group previously identified Notch upregulation as a driver of EAC development.^32^ We also found that overexpressing Notch in the BE/EAC mouse model upregulated Wnt and downstream target genes. Similar clusters were noted by Guo et al. in analyses of three existing transcriptomic datasets from EAC tumors^16^; using the list of genes from that study that distinguished the clusters, we reproduced the findings in our BE patients. Jammula et al. performed an integrated analysis of EAC tumors as well as paired BE and identified four clusters (termed “subtypes”) based on DNA methylation profiles, with subsequent incorporation of genomic and transcriptomic data.^33^ It is unclear how translatable these findings are to the current study, as these clusters were based on DNA methylation profiles.

After adjusting for key clinical covariates, we found very few significant alterations in gene expression with progressive stages of neoplasia. We observed upregulation of *LAIR2*, which is postulated to represent a marker of T cell exhaustion in tumors^34^ and perhaps reflects changes to the immune microenvironment in BE-associated neoplasia.^17,18^ Recent transcriptomic analyses from 16 EAC resections simulating bulk RNA-Seq using single cell sequencing data found that BE tissue without dysplasia clustered with dysplasia and EAC.^35^ Only when analyses were stratified by cell type were gene expression differences apparent across stages of neoplasia. Interestingly, *LAIR2* was one of the genes found to be upregulated in CD8+ T cells in dysplasia and EAC, suggesting that this may represent a novel immune marker in BE-associated neoplasia.

There were numerous strengths to the current analyses. This was a large, well-characterized population of BE patients with and without associated neoplasia. Stratified enrollment of controls who were and were not taking PPIs allowed for additional insights into the potential effects of acid exposure. Comprehensive profiling was performed of multiple specimen types, incorporating information on composition of refluxate bile acids and bacterial communities and relating these to tissue gene expression, with many analyses adjusted for key clinical variables. Central pathology review was performed to rigorously categorize patients’ associated histology.

There were certain limitations to the study, as the cross-sectional design limits any inferences that can be drawn regarding causality. However, despite this the lack of deconjugated bile acids in refluxate strongly suggests that there is little bacterial metabolism of luminal bile acids in the upper GI tract. While we quantified total and individual bile acids from refluxate, we were unable to capture cumulative bile acid exposure in the esophagus over time. We opted for 16S rRNA gene sequencing over metagenomic sequencing due to the low biomass nature of the samples, and this limited our ability to derive functional characteristics of the bacterial populations themselves. Transcriptomic analyses were only performed on a subset of patients, potentially limiting our power to detect additional important findings. The use of bulk RNA-Seq as opposed to single cell sequencing may have masked additional relationships between bile acids, bacteria, and gene expression.

In conclusion, in this large study of patients with and without BE with comprehensive characterization of reflux bile acids, the microbiome, and the transcriptome, patients with BE had a distinct refluxate bile acid composition characterized by increased total and secondary bile acids. BE patients also had a distinct lower esophageal microbiome, possibly shaped by refluxate bile acids and influenced partly but not completely by PPIs. Both refluxate bile acid and lower esophageal bacterial composition were associated with numerous gene expression alterations that may contribute to progression to EAC. Interestingly, two BE gene expression clusters were identified and not associated with histology or bile acid or microbiome composition; the clinical significance of these remains to be determined. Future studies are warranted to determine whether therapies aimed at modifying the bile acid-bacteria axis in the upper GI tract can impact risk of development of EAC.

## Supplementary Material

Supplementary Table 2.docx

Supplementary Figures.docx

Supplementary Methods.docx

Supplementary Table 1.docx

Supplementary Table 3.xlsx

## Data Availability

Data for bacterial 16S rRNA gene sequencing are submitted to the NCBI Sequence Read Archive (SRA) under accession number PRJNA1136523. Data for human RNA sequencing are submitted under accession number PRJNA1063201. Code used for data analysis can be found under https://github.com/ctanes/betrnet_b2_paper.

## Abbreviations

BE: Barrett’s esophagus
ND: nondysplastic BE
IND: indefinite for dysplasia
LGD: low grade dysplasia
HGD: high grade dysplasia
EAC: esophageal adenocarcinoma
GERD: gastro-esophageal reflux disease
KEGG: Kyoto Encyclopedia of Genes and Genomes
PPI: Proton pump inhibitor
TDCA: Taurodeoxycholic acid
GDCA: Glycodeoxycholic acid
