# Supplementary material for: Increased reflux secondary bile acids are associated with changes to the microbiome and transcriptome in Barrett’s esophagus
Source: Gut Microbes. 2025 Aug 22;17(1):2545420. doi: 10.1080/19490976.2025.2545420 (PMC12377100; doi:10.1080/19490976.2025.2545420)
Supplement: Supplementary Table 2.docx [file KGMI_A_2545420_SM8552.docx]

| **Supplementary Table 2.** Patient and endoscopic characteristics comparing the three gene clusters. | | | | | |
| --- | --- | --- | --- | --- | --- |
|  | **Cluster 1 (n=48)** | **Cluster 2 (n=21)** | **Cluster 3 (n=40)** | **p-value, Cluster 1 vs Cluster 2** |  |
| ***Patient characteristics*** |  |  |  |  |  |
| Age, years: mean (SD) | 66.8 (±10.0) | 65.2 (±11.7) | 51.4 (±14.8) | 0.6 |  |
| BMI: mean (SD) | 29.6 (±4.9) | 32.0 (±6.6) | 30.4 (±7.0) | 0.1 |  |
| Male sex: n (%) | 39 (81%) | 14 (67%) | 22 (55%) | 0.2 |  |
| PPI use: n(%) | 48 (100%) | 20 (95%) | 26 (65%) | NA |  |
| Aspirin use: n (%) | 18 (38%) | 10 (21%) | 10 (25%) | 0.44 |  |
| Statin use n(%) | 26 (54%) | 14 (67%) | 8 (20%) | 0.43 |  |
| Reflux n(%) | 46 (96%) | 20 (95%) | 28 (70%) | 1.0 |  |
| Ever smoker: n (%) | 27 (56%) | 13 (62%) | 14 (35%) | 1.0 |  |
| Family history of BE or esophageal cancer: n (%) | 15 (31%) | 6 (29%) | 4 (10%) | 1.0 |  |
| ***Endoscopic characteristics*** |  |  |  |  |  |
| Histology: Cntrl:NDBE:IND:LGD:HGD:EAC | 1:21:7:8:8:3 | 1:11:0:3:3:3 | 35:4:1:0:0:0 | 0.4 |  |
| Hiatial hernia size: median (IQR) | 3(IQR 2-4.75) | 3(IQR 2-4) | - | 0.5 |  |
| Length of contiguous BE (C): median (IQR) | 2(IQR 0-6) | 0 (IQR 0-1) | - | <0.001 |  |
| Length of contiguous BE (M): median (IQR) | 5(IQR 3-8) | 3 (IQR 2-4) | - | <0.001 |  |

**Wilcoxon or Fisher exact p-values reported for difference between pairs of clusters*
