# Supplementary material for: Increased reflux secondary bile acids are associated with changes to the microbiome and transcriptome in Barrett’s esophagus
Source: Gut Microbes. 2025 Aug 22;17(1):2545420. doi: 10.1080/19490976.2025.2545420 (PMC12377100; doi:10.1080/19490976.2025.2545420)
Supplement: Supplementary Figures.docx [file KGMI_A_2545420_SM8551.docx]

**Supplementary Figures**

**Supplementary Figure 1.**  Flow diagram of patients included in the study.

**Supplementary Figure 2.** Graphical depiction indicating sample types collected and analyzed (blue) for each subject enrolled.

**Supplementary Figure 3.** A) Correlations between individual bile acid levels and global measures of bacterial composition at different sampling sites, comparing BE with controls on and off of PPIs. B) Correlations between individual bile acid levels and bacterial genera with significantly altered relative abundance in BE and/or across stages from BE to EAC. * p<0.05, ** p<0.01, ***p<0.001

**Supplementary Figure 4.** Using the gene list from Guo et al.^24^, Clusters 1 and 2 from our own analyses again clustered separately and independent of associated histology.
