# Supplementary material for: Increased reflux secondary bile acids are associated with changes to the microbiome and transcriptome in Barrett’s esophagus
Source: Gut Microbes. 2025 Aug 22;17(1):2545420. doi: 10.1080/19490976.2025.2545420 (PMC12377100; doi:10.1080/19490976.2025.2545420)
Supplement: Supplementary Methods.docx [file KGMI_A_2545420_SM8550.docx]

***Supplementary Methods***

***Study Population and Data Collected***

Any adult patient scheduled to undergo upper endoscopy for clinical indications was potentially eligible for the study. Patients were >18 years old, and for BE patients, had histologically confirmed BE ≥2 cm in length and took proton pump inhibitors (PPIs) at least daily for 3 months prior to enrollment. Patients without BE were enrolled stratified 1:1 based on current PPI use. Patients with a history of gastric cancer or esophageal squamous cell cancer or gastric or esophageal surgery, who used antibiotics or systemic immunosuppressants within three months prior to the date of endoscopy or had known untreated esophageal stricture or uninvestigated dysphagia were excluded. Inability to provide informed consent was also an exclusion criterion, and vulnerable populations were not recruited for this study.

Data were collected on demographic and clinical information including medical history, history of prior endoscopies and associated histology, family history of BE or esophageal cancer, medication use, and smoking history. In addition, anthropometric measures were recorded to calculate body mass index (BMI) and waist-to-hip ratio (WHR).

During the endoscopy, BE length, hiatal hernia size, and presence of focal lesions within the BE segment were noted, and biopsies were taken per standard of care. Patients were classified by the highest degree of neoplasia and categorized as one of the following: no BE, nondysplastic BE (ND), indefinite for dysplasia (IND), low grade dysplasia (LGD), high grade dysplasia (HGD), or adenocarcinoma. All patients with EAC in this study had intramucosal (T1a) adenocarcinoma. Biopsies were interpreted by pathologists at the corresponding institution where the endoscopy was performed and also reviewed by a central pathologist (ADP). If there was disagreement between the local and central pathologists’ readings, the final diagnosis was determined by consensus review with a second central pathologist (SML). If the diagnosis was indefinite for dysplasia, slides were re-reviewed to determine a consensus diagnosis. If a subject had a history of a higher degree of neoplasia detected on a prior endoscopy (compared to the study endoscopy), the subject was categorized by the historical findings if these biopsies had been reviewed by an expert GI pathologist at one of the study sites.

Patients provided written informed consent prior to enrollment in the study. Patients and Public were not involved in the study design, choice of outcome measures, or recruitment.

***Biospecimens***

Multiple biospecimens were collected while patients were in the fasting state. Just prior to the endoscopy, saliva and oral rinse as well as blood were collected. Sterile water was collected and stored as a negative control for the oral samples. Prior to starting the endoscopy, an environmental negative control was collected by passing a sterile brush catheter through the endoscopic channel. During the endoscopy, 5 mL of gastric fluid was suctioned for subsequent bile acid profiling. Additional biopsies were taken for gene expression analyses from the mid-BE segment (from BE patients) or the gastric cardia within 1 cm of the squamo-columnar junction (from non-BE patients), avoiding any focal lesions. For tissue microbiome analyses, brushings were taken from the squamous esophagus (3 cm proximal to the squamo-columnar junction) and from BE (or cardia in control patients). The biospecimens were stored at -80˚ C.

***Bile acid profiling***

Gastric aspirate samples were thawed on wet ice, briefly vortexed to homogenize, and centrifuged at 10,000x g for 1 minute. 25 µL of the supernatant was transferred to a Total Recovery Vial (Waters Corporation, Milford, MA) and loaded into an autosampler at 4^o^C for analysis. Samples were analyzed on an Acquity uPLC with a Cortecs UPLC C-18+ 1.6 mm 2.1 x 50 mm column and a QGa single quadrupole mass detector.^11^ The flow rate was 0.8 mL/min, the injection volume was 4 uL, the column temperature was 30^o^C, the sample temperature was 4^o^C, and the run time was 4 min per sample. Eluent A was 0.1% formic acid in water; eluent B was 0.1% formic acid in acetonitrile; the weak needle wash was 0.1% formic acid in water; the strong needle wash was 0.1% formic acid in acetonitrile, and; the seal wash was 10% acetonitrile in water. The gradient was 70% eluent A for 2.5 minutes, gradient to 100% eluent B for 0.6 minutes, and then 70% eluent A for 0.9 minutes. The mass detection channels were: +357.35 for chenodeoxycholic acid and deoxycholic acid; +359.25 for lithocholic acid; -407.5 for cholic acid; -432.5 for glycolithocholic acid; -448.5 for glycochenodeoxycholic and glycodeoxycholic acids; -464.5 for glycocholic acid; -482.5 for taurolithocholic acid; -498.5 for taurochenodeoxycholic and taurodeoxycholic acids; and -514.4 for taurocholic acid. Samples were quantified against standard curves of at least five points run in triplicate. Standard curves were run at the beginning and end of each metabolomics run. Quality control checks (blanks and standards) were run every eight samples.

***Microbiome analyses***

DNA was extracted from samples using the Qiagen DNeasy PowerSoil Pro kit. Extracted DNA was quantified with the Quant-iT PicoGreen Assay Kit. Barcoded PCR primers annealing to the V1-V2 region of the 16S rRNA gene was used for library generation. PCR reactions were carried out in duplicate using Q5 High-Fidelity DNA Polymerase (NEB, Ipswich, MA). Each PCR reaction contained 0.5 uM of each primer, 0.34 U Q5 Pol, 1X Buffer, 0.2 mM dNTPs, and 2.5 ul DNA in a total volume of 25 ul. After amplification, PCR reactions were pooled and then purified using a 1:1 volume of SPRI beads. DNA in each sample was then quantified using PicoGreen and pooled in equal molar amounts. The resulting library was sequenced on the Illumina MiSeq using 2x250 bp chemistry. Extraction blanks and DNA free water were subjected to the same amplification and purification procedure to allow for empirical assessment of environmental and reagent contamination. Positive controls, consisting of eight artificial 16S gene fragments synthesized in gene blocks and combined in known abundances, were also included.

Sequence data were processed using QIIME2 version 2019.7.^12^ Read pairs were processed to identify amplicon sequence variants (ASVs) with DADA2.^13^ Taxonomic assignments were generated by comparison to the Greengenes reference database^14^, using the naive Bayes classifier implemented in scikit-bio.^15^ A phylogenetic tree was inferred from the sequence data using MAFFT.^16^ Similarity between samples were assessed by weighted and unweighted UniFrac distances.^17, 18^

***Tissue gene expression analyses***

Esophageal tissue gene expression analyses were performed by bulk RNA sequencing (RNA-Seq). Esophageal biopsies were homogenized in Qiazol on Tissuelyser II (Qiagen) at frequency 25/s for 2 minutes, twice. Total RNA was then purified using the miRNeasy micro kit (Qiagen) following the kit protocol. RNA was eluted with 30µl RNase-free water. Quantitation was done by Nanodrop and Bioanalyzer. Poly-A pull-down was performed to enrich mRNAs from total RNA samples, then library construction was performed using Illumina TruSeq chemistry. Libraries were then sequenced using Illumina NovaSeq 6000 using paired-end 100bp chemistry. Fastq files were generated using bcl2fastq2 (version 2.19), coupled with adapter trimming. Reads were mapped to the human transcriptome (GRCh38) using the pseudoalignment software kallisto (0.44.0).^19^ Analyses were restricted to those with RNA Integrity Number (RIN) values greater than 5.
