# Supplementary material for: Increased reflux secondary bile acids are associated with changes to the microbiome and transcriptome in Barrett’s esophagus
Source: Gut Microbes. 2025 Aug 22;17(1):2545420. doi: 10.1080/19490976.2025.2545420 (PMC12377100; doi:10.1080/19490976.2025.2545420)
Supplement: Supplementary Table 1.docx [file KGMI_A_2545420_SM8549.docx]

**Supplementary Table 1.** Indications for upper endoscopy among control patients included in the analyses (n=52). Note: some patients had more than one indication for endoscopy.

| **Indication** | **N** |
| --- | --- |
| Abdominal pain/dyspepsia | 22 |
| GERD | 18 |
| Celiac disease | 5 |
| Anemia/GI bleed | 4 |
| Diarrhea | 3 |
| Other* | 7 |

*One patient each for the following indications: duodenal polyp, dysphagia, weight loss, history of ulcer, cirrhosis, hernia, gastritis
